# Integration of a Theoretical Mechanism Into an Intervention to Prevent Elder Mistreatment in Family Caregiving

**DOI:** 10.1093/geroni/igaf122.1423

**Published:** 2025-12-31

**Authors:** Kylie Meyer, Jaclene Zauszniewski, Wenxing Wei, Zachary Gassoumis, Alexander Gonzales, Elliane Irani, Jeanine Yonashiro-Cho, Donna Benton

**Affiliations:** Case Western Reserve University, Cleveland, Ohio, United States; Case Western Reserve University, Cleveland, Ohio, United States; Case Western Reserve University, Cleveland, Ohio, United States; UTHealth Houston, Houston, Texas, United States; University of Southern California, Los Angeles, California, United States; Case Western Reserve University, Cleveland, Ohio, United States; University of Southern California, Los Angeles, California, United States; University of Southern California Leonard Davis School of Gerontology, Los Angeles, California, United States

## Abstract

Approximately half of family caregivers of persons living with Alzheimer’s Disease and Related Dementias (AD/ADRD) engage in elder mistreatment (EM), with psychological EM being among the most common types. The KINDER (Knowledge and Interpersonal Skills to Develop Enhanced Relationships) intervention aims to prevent psychological EM and support high quality care by enhancing caregiving relationships. Initially, KINDER lacked a clearly defined theoretical framework, a known barrier to intervention translation. This study describes the iterative integration of Resourcefulness and Quality of Life Theory© (RQoLT) into KINDER to establish a plausible theory-based mechanism of change. Two studies were conducted. Study 1 involved qualitative interviews with seven caregivers who participated in the first KINDER intervention. Thematic analysis identified the use of personal resourcefulness skills, and, to a lesser extent, social resourcefulness skills. Based on these findings, KINDER was refined to include small group discussions to promote the use of social resourcefulness, and to emphasize the development of each type of resourcefulness. Study 2 tested the revised intervention with 71 caregivers, using qualitative interviews and surveys to assess the applicability of RQoLT. Caregivers reported using personal and social resourcefulness skills to improve care relationships and prevent EM. The revised intervention demonstrated continued applicability of RQoLT, with caregivers showing increased reliance on personal resourcefulness skills. Findings suggest RQoLT is a plausible theory of intervention for KINDER, supporting the development of such skills to prevent psychological EM and promote high-quality caregiving. Further research is needed to rigorously test resourcefulness as a mechanism and identify effective intervention components.

